# Students' perceptions of the impact of assessment on approaches to learning: a comparison between two medical schools with similar Curricula

**DOI:** 10.5116/ijme.4ddb.fc11

**Published:** 2011-05-27

**Authors:** Hanan M. Al Kadri, Mohamed S. Al-Moamary, Mohi Eldien Magzoub, Christopher Roberts, Cees van der Vleuten

**Affiliations:** 1 College of Medicine, King Saud bin Abdulaziz University for Health Sciences, Saudi Arabia; 2 Sydney Medical School-Northern, University of Sydney, Australia; 3 Department of Educational Development and Research, University of Maastricht, the Netherlands

**Keywords:** Assessment methods, learning approaches, cultural differences

## Abstract

Objectives: The aim of the study was to investigate students? perceptions of assessment and the resulting learning styles.

**Methods:**

Qualitative semi-structured interviews were conducted with 14 students and 8 clinical supervisors from Sydney Medical School and 12 students and 13 clinical supervisors from King Saud bin Abdulaziz University. Both institutions have similar curricula but a different assessment approach. The interviews were transcribed and analyzed using thematic analysis. Interview transcripts were stored and analyzed using ATLAS.ti.

**Results:**

Three themes emerged from analyses of the interviews: the function of assessment, learning outcomes and, finally, authentic assessment in the clinical environment. A model is presented to show the relationship between contextual and different personal factors and students’ perceptions of the impact of assessment on learning styles.

**Conclusions:**

Cultural differences and emotions can affect students’ perceptions of assessment and learning styles. A combination of formative and summative assessment based on learning objectives is required. This combination should take into consideration students’ cultural background, values and the implemented education system. This balance should be sufficient to motivate students in order to maintain their focus and attention, and reduce the potential negative impacts of a hidden curriculum. The experience of authentic assessment was a powerful motivator for students’ approaches to learning.

## Introduction

It is well known that assessment is one of the most important factors affecting students’ approaches to learning.^1^^-^^4^ Although many researchers emphasize this relationship,^4^^-^^8^ it continues to be poorly understood particularly with regard to the following aspects: 1) the persistent incongruence between curricular and assessment objectives; 2) the purpose of assessment methods (formative/summative); and 3) the effect of personal influences, such as students’ expectations for specific courses, academic discipline, prior education, age and gender, and cultural influences.^9^Crooks et al.^10^ warned against the possible incongruence between academic objectives as intended by the curriculum and the objectives defined through the assessment process. Synchronization between these two types of objectives is called constructive alignment. When constructive alignment is achieved, it is assumed to be conducive to learning. Biggs^11^ and Ramsden^2^^,^^12^ have described the interactive relationship among student factors, teaching context, the on-going approaches to a particular task and student learning outcomes. Through teaching and learning with consideration to this interactive relationship, learners will usually learn what should be learned. Therefore, one of the consequences of curriculum misalignment is that repeated discrepancies between what students perceive that they need to know for assessment purposes and the stated course objectives can potentially lead to a local culture, whereby a hidden curriculum^13^ is created. Hafferty^13^ defined the hidden curriculum as "a set of influences that function at the level of organizational structure and culture.” Its nature depends on the students’ own interests, supervisors’ interests, and even students’ personal speculations about what might be in their summative assessments.^6^In this study, the term "summative assessment" refers to an assessment performed to assign students a course grade, whereas the term "formative assessment" refers to an ungraded assessment that carries meaningful information as an educational tool to aid students' learning. Existing research on the effects of summative and formative assessment on students’ learning strategies is unequivocal. Whilst some researchers claim that formative assessment is more effective than summative assessment in producing deep learning strategies,^6^^, ^^14^ others disagree.^15^^, ^^16^ Researchers have concluded that feedback and formative assessment produce the most powerful effect on student achievement.^16^^, ^^17^Formative assessment appears to play a larger role in increasing student achievement than does a reduction in class size or an increase in teachers' content knowledge.^18^ On the other hand, summative assessment is a proven way of eliciting evidence of student achievement and discriminating between students of differing abilities.^19^^, ^^20^ therefore similar to formative assessment, summative assessment may prompt feedback from faculty that promotes students’ learning.The third area of research where there is a lack of clarity is the effect of personal influences on students’ approaches to learning. Students enter a course or a program with specific intentions about the study strategies that they are likely to employ.^7^ These strategies are mediated by differing personal and contextual influences and the different ways by which students perceive assessment and its demands.^6^^, ^^7^ Vermunt^21^ tried to clarify the relationship between the way students learn and personal and contextual variables. He found that educational contexts like the way the learning environment is structured and personal factors; such as academic discipline, prior education, age and gender, had an effect on students’ learning patterns. For example, older or more experienced students showed greater ability to differentiate between various learning strategies than younger or less experienced students.^22^Furthermore, culture is a personal factor that encompasses students’ beliefs, behaviors, attitudes, and practices that are learned, shared and passed on.^23^ Students’ sense of a “cultural identity”^24^ is derived from a complex mixture of cultural, gender, social, economic, religious, and political affiliations. Little is known about how these different cultural factors might influence the way students perceive their assessment and affect their study strategies. Due to this inconsistency in our understanding of the effects of different personal and contextual factors on students’ perceptions of the implemented assessment and their approaches to learning, further research is needed.We were in a unique position to address our research aim by comparing students’ perceptions of assessment and the students’ resultant learning strategies in two programs in two different countries, with differing cultural values. At the time of the study, the King Saud Bin Abdulaziz University for Health Sciences, College of Medicine (KSAU-HS, COM), Riyadh, Saudi Arabia had implemented a four-year graduate entry Problem-Based Learning (PBL) curriculum derived from that of Sydney Medical School (SMS). However, both had different assessment processes implemented during the third and fourth year of the curriculum (clinical years).The assessment process during the clinical years in SMS was characterized by different requirements for each clinical block. During these blocks, students rotate in different clinical attachments where at the end of each they must complete a formative self-assessment form. This self-assessment was done in parallel with supervisors’ formative assessment and feedback. Students were also required to complete a variety of summative assessments, formative clinical exercises and online assessments that were differed from block to block. A multiple-choice question (MCQ) barrier exam was conducted by the end of the third year, which the students had to pass in order to progress to the final year. Based on their performance in their barrier exam, students were provided with summative feedback indicating their grades in each discipline and their standing in relation to their peers. Finally, a summative long case must be completed during the last year of the curriculum.The assessment program for the third and fourth year of the curriculum at KSAU-HS, COM was block-based and was similar from block to block. Students must pass all clinical blocks prior to their graduation. In each clinical block, students rotate in different clinical attachments where at the end of each they must complete formative self-assessment form paralleled with supervisors’ formative assessment and feedback. Moreover, in each block, students’ assessment was divided into two main parts. The continuous assessment of students’ performance accounted for 40% of the final grade, and the final block examination accounted for the other 60%. In summary, the third and fourth years of the curriculum assessment program of KSAU-HS, COM in comparison to SMS assessment program was characterized by 1) uniformity from block to block 2) less formative assessment 3) more frequent structured summative assessments and 4) no barrier exams.The research aim of this study was to provide a theoretical insight into the interaction of different personal and contextual factors on students perception of the faculty implemented assessment in a clinical context and to understand their impact on students’ resultant approaches to learning.

## Methods

### Study Setting

In both study settings, KSAU-HS, COM and SMS, third- and fourth-year students rotate through different clinical blocks, including medicine and surgery and specialist blocks. In each clinical block, students will join different clinical attachments supervised by clinical supervisors. This stage of the training program utilizes case based learning to augment direct patient contact.

### Study Design

A qualitative approach using thematic analysis^25^ was used to generate a rich understanding of the full range of opinions and experiences of students when they are exposed to the implemented assessment. Our assumption was that students of different cultural background were influenced in their approach to learning by different personal and contextual factors. In interpreting our data we used a theoretical framework based on the work of Biggs^11^ and Ramsden^2^^, ^^12^ describing the interactive relationship among student factors, teaching context, the on-going approaches to a particular task and student learning outcomes.

### Study Population

The study participants were students who were in the last two years of the curriculum. This convenience sampling was undertaken to gain students’ common experiences and perceptions of the various methods of assessment implemented during this phase of the curriculum. In order to provide a richer insight into the contextual factors associated with working in a clinical placement, we also interviewed students’ clinical supervisors. Accordingly, our data set incorporated the perspective of the experienced clinicians in charge of implementing the formal process of teaching and assessment.At the time of the research conduction, KSAU-HS, COM was accepting only male students into the Medical program. All eligible students (61 students) and clinical supervisors (fifty six supervisors) were invited to participate in the study through an e-mail announcement and direct contact. Twenty eight students and all supervisors agreed to participate in the semi-structured individual interviews. We interviewed twelve students and thirteen supervisors, after which our analysis revealed data saturation.In SMS, all third- and fourth-year students and their supervisors were invited to participate in the research through e-mail announcements. Those who accepted were called for interviews (fourteen students; eight males and six females, and eight supervisors) were interviewed after which our analysis revealed data saturation. The first author and a research assistant conducted interviews. At the time of the interviews, the first author was not involved in the academic experiences of the students participating in the study.

### Data Collection

Semi-structured individual interviews and open-ended questions were conducted with students and supervisors. We explored students’ experience of the curriculum, its learning objectives alignment with students learning activities and the given assessment. Furthermore, we explored students’ experiences of feedback and the different assessment implemented with a particular focus on the ways in which students’ practice had influenced their learning approaches. Each interview lasted from 30-45 minutes. Interviews were recorded on audiotape and transcribed verbatim.

### Analysis

Interview data was examined in-depth aiming to obtain the emerging themes. Initial coding revealed a number of basic themes that were arranged to form organizing themes. Subsequently, organizing themes were iteratively discussed between authors and were renegotiated when differences existed. After further analysis, the organizing themes were condensed into the three global themes discussed in this paper.^25^ The analysis of the transcriptions of the interviews were managed using Atlas.ti (Version 5.2).

### Ethical Approval

The approvals of the University of Sydney Human Research Ethics and KSAU-HS were obtained prior to conducting the research.

## Results

A theoretical insight is presented that illustrates first, how students with different personal characteristics including their cultural backgrounds are influenced by contextual assessment related factors. Second, how this complex interaction affects their learning approaches. Our results are organized into three main themes: 1) students’ personal perceptions of assessment function; 2) students’ perceptions of learning outcomes; and 3) the student experience of authentic assessment in the clinical environment. We present these themes with illustrative quotes from students (S) and supervisors (T) from either SMS (U) or KSAU-HS (K).

### Students Personal Perceptions of Assessment Function

#### Summative Assessment

Summative assessment was appreciated by all students due to its ability to provide students with a clear idea of their progress. It was perceived as a major factor in stimulating students to study more, exert more effort to pass an exam or get a higher mark; it can thus be considered as a stimulus to effort and achievement, as well as being a motivation strategy for study.

*“If your exam counts and there will be a grade next to your name, you are going to be more serious when preparing for the exam”* (SK).

The Saudi students particularly noted that this phenomenon related to their prior educational experience and the inherent importance given to summative assessment during their high school study and previous university degrees:

*“…we care about our marks, we study for the marks, …we were raised like this. When we joined here…., problem based learning did not change us. We still focus on the marks….even our supervisors they focus mainly on our marks…”* (SK).

Supervisors likewise perceived summative assessment as a stimulus for hard work, improved clinical performance, enhanced patients’ safety and was a successful method in motivating students to increase their effort and improve their achievement. They believed that summative assessment could stimulate the development of better approaches to learning. Supervisors believed that students who pass their summative assessment would become better future doctors and therefore they recommended including this type of assessment in the curriculum assessment strategy:

*“if students can’t pass their summative assessment, that means they are not really suitable to be doctors”* (TU).

Although summative exams lead to increased anxiety among the students, some of them were able to cope effectively. Our data suggests that coping methods could have been influenced by cultural background since students in each of the schools used different coping strategies. Stressful situations led KSAU-HS, COM students to resort to sporadic, patchy, superficial reading and to study the information they thought was important or might appear in the exam without going into the depth of the subject. These practices represent a superficial approach to learning:

*"We have to study for the exam, we have to read for the clinical sessions; how can I get time in between? I am always stressed, very stressed"* (SK*).*

Similarly, SMS students experienced stress when undergoing summative assessment, but they perceived this stress positively. It stimulated them to work hard, helped them develop deeper approaches to learning and enhanced their performance on exams:

*“…. the stress…. I like the stress…… stress helps. It motivates me. It is a little bit stressful but I think this is part of our learning”* (SU).

We note that on top of the cultural differences, KSAU-HS, COM students’ stress might have been related to the fact that they faced more frequent summative assessments. It may also be linked to their anxiety regarding future opportunities to enroll in post-graduate study, obtain a scholarship or even get a job, all of which could be determined by their accumulative assessment marks. On the other hand, SMS students were more confident of progressing as long as they passed all the assessment tasks.

Neither cultural differences nor differences in the implemented methods of assessment affected supervisors’ perception of both students’ anxiety and the purpose of summative exams. All supervisors considered this stress as a positive element that improves student learning, prepare them for patients care, future work challenges and helps students to practice effort and achievement motivation strategies while coping with this stress:

*“So it is definitely the most stressful experience for students, but you know life is full of stress and if we just try to remove all stress, students will not be prepared to deal with real life stress.”* (TU)

Our data suggest that the learning approaches used by KSAU-HS, COM students to prepare for their summative assessment were generally more superficial and geared toward short-term results than were the approaches of the students at the SMS. These differences were a result of multiple personal and contextual factors, including different students’ ability to cope with stress in the two study settings and the more frequent summative assessments faced by the KSAU-HS, COM students.

#### Formative Assessment

When the supervisors used formative assessment properly, it helped students to identify learning objectives and to improve their study strategies. This resulted in an improved ability of students to diagnose patients’ diseases, apply theoretical knowledge to patient care, and plan appropriate management strategies. Therefore, formative assessments stimulated meaningful and multifaceted learning. However, students in SMS were more capable of accommodating negative feedback and perceiving it positively than were KSAU-HS, COM students. The SMS students were more enthusiastic about receiving and using direct verbal feedback:

*“I like negative feedback, I think it is good. To me it just tells me to go and read more… you need to be reminded to take care”* (SU).

It appears that cultural values and preferences may have contributed to the KSAU-HS, COM students’ perception of negative feedback and their ability to accommodate it. They considered negative feedback as criticism carrying no constructive value:

*“negative feedback puts students under more stress. You work hard for long time and at the end your performance is assessed to be poor…” (*SK).

Students and supervisors in both settings disagreed about the suitable frequency of formative assessment and feedback. The recommended frequency ranged from once every few months to twice every week:

*“A formative exam may be once every two blocks, you know just to get people to know what the barrier exams are going to be like.”* (SU) and *“at least once or twice a week and not less than that.”* (SU).

This wide range maybe attributed to the differing perceptions of the appropriate amount of time and effort necessary for completion and evaluation of assignments.

### Students Perception of Learning Outcomes

Students considered assessment fair and a stimulus to deep approach to learning when it was tailored to curriculum objectives. When the formulated objectives were neither specific nor precise, students directed their studying towards selected objectives. They used their own opinions, exam experiences, feelings and speculations in creating their own curriculum. The tendency to create a hidden curriculum^13^ was more prevalent among the SMS students, which may reflect a better understanding of the curriculum implementation and assessment process and greater academic independence. The more formative emphasis of the SMS assessment program may have contributed to the students’ behavior. On the contrary, the more summative emphasis of KSAU-HS assessment program made the students more hesitant to create their own study agenda or a hidden curriculum:

*“I didn’t find the objectives very useful. I’ll never really study in that way. I am happier to just focus mainly on the clinical stuff….so it’s more of personal preferences.”* (SU) and *“Usually I start by looking into the objectives and I make sure that when I study I read these points specifically... I found that quite helpful….”* (SK).

The presence of broad, imprecise objectives across the curriculum have resulted in variations in students’ understanding of their assignments and therefore variations in their approaches to learning. Students who studied based on the curriculum objectives have opted to study as much as possible of the many topics covered in their objectives without focusing on their importance, relevance or linking it to patients’ management. That lead to superficial approach to learning. Others have opted to create their own objectives based on preferences, senior advises or what they have observed as important information during their direct patients contact. Therefore, they studied based on these selected objectives and linked it to patients’ care resulting in deep approach to learning for selected topics and superficial approach to learning for others. These approaches variations in response to broad imprecise objectives did not differ between the two studied groups of students with higher tendency to create a hidden curriculum between SMS students:

*“…the problem I have found is that there is no guide to how much you need to know on that subject. It is too broad…you can read a textbook or you can read a very brief definition...it usually depends on the students themselves how much they decide to study “(*SU*) and “To read based on the objectives….that*

*will take a lot of time. What I usually do, is to look into the information given to us…and just study this information and expand on the points that I need…”*(SU).

What made the hidden curriculum even more prevalent is students’ perception of their learning outcome and its relation to their assessment. Students stressed on the role of personal values and preferences on their learning approaches. They thought that safe patients care and good practice are more motivating for quality studying compared with faculty assessment. This perception was not affected by the different cultural and contextual differences between the two studied groups:

*“I have a certain expectation of myself; I would perform indeed as a safe practitioner, and to be a safe practitioner. I need to know my limits and I need to know when to ask for help. I do not think the assessments we have really reinforce these points”* (SU).

Direct patients contact and the authentic assessment in the work place were connected to a better learning approach, which directs us to our third identified theme.

### Student Perception of Authentic Assessment in the Workplace

All students in both settings preferred and appreciated observed clinical assessment. They perceived it as reflecting their future performance and patient-care capabilities. Constructive and direct supervision helped students integrate clinical knowledge into practice, summarize patients’ histories and solve patients’ problems. When supervisors acted as role models, their methods of coaching and guiding students, experience and commitment to clinical teaching were crucial factors in enhancing the quality of learning and improving students’ performance on exams.Direct patient encounters improved students’ performance and confidence in performing clinical examinations and enhanced student learning. It helped students in directing their efforts towards solving their patients’ problems and encouraged clinical work.However, in both study settings, there were variations in students' responses to their clinical experiences. It appeared that, in addition to direct patient encounters, general and personal factors are needed to enhance students’ motivation. Moreover, supervisors’ investment of time, interest and teaching skills were important enhancements. Supervisors felt that an appropriate balance between clinical tasks and teaching motivated them to involve students in patient management and to consider them as part of the managing team. When the clinical supervisors had more teaching time and experience, they were more capable of stimulating students’ interest in clinical training and fostering favorable learning conditions:

*“The most important trigger for students to learn is their new experience in the hospital. The resulting excitement…stimulates them to do more reading and preparation for assessment”* (TK); *“I’m not sure that all students have the same preferences. There is a student who is supposed to be my partner in this rotation; he has not been seen for the last two weeks. Obviously that person has not turned out for anything at all.”* (SU).

## Discussion

**Figure 1:**
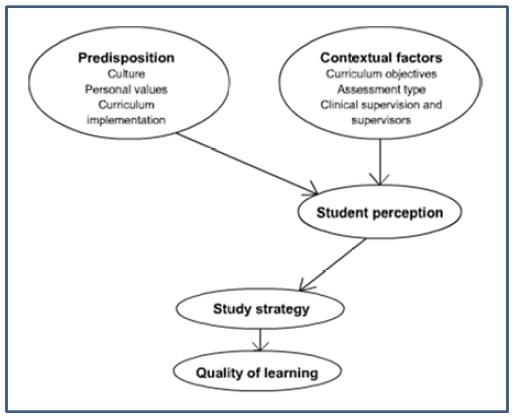
The effect of different contextual factors and various predispositions on students’ perception of their assessment and learning

Our study has provided an insight into the interactive relationship between students’ personal values, cultural factors, teaching context, their perceptions of assessment tasks and the students’ resultant learning approaches as they progressed through their clinical placements. Important factors affecting students’ perceptions of their assessment included the type of assessment used (summative or formative), their perception of curriculum objectives and learning outcome, and the presence and nature of authentic clinical assessment with the involvement of clinical supervisors. Variations in students’ study strategies were influenced by; firstly different contextual processes of curriculum (including assessment) implementation; and secondly, by students’ cultural backgrounds and personal values and preferences. These different perceptions have resulted in differences in students’ learning approaches (Figure 1).

The cultural differences between the two studied groups appears to have influenced their perception of learning and their approach to their studying.^23^ This resonates with previous research on cross-cultural learning styles and learning behaviors which has recommended that students’ approaches to studying must be interpreted in the educational, institutional and cultural contexts within which the study occurs.^29^ This partly reflects prior educational experience and the prevailing educational systems in differing cultures. For example, in one study, Asian students tend to have difficulty adjusting to an educational environment that is characterized by independent learning and less instructor supervision and guidance.^30^ Saudi students similarly are thought to have had a teacher-oriented, lecture-style learning environments^31^ prior to medical school.In this study, both studied groups acknowledged the importance of summative assessment as a motivator for hard work. However, in contrast to SMS students, the KSAU-HS, COM students perceived summative assessment as a stressful and anxiety-provoking experience that led them to engage in sporadic and superficial reading. Therefore, the summative system may have both positive and negative influences on students’ learning. These influences depend upon students’ perceptions. The behavioral symptoms displayed by the KSAU-HS, COM students during preparation for and completion of summative exams might be what is known as “test anxiety”. Based upon the literature, about 10% of students suffer from test anxiety, which compromises their performance and emotional well-being.^32^^, ^^33^ This problem is not specific to KSAU-HS, COM students. Severe test anxieties have been reported for medical trainees in many countries with different social and financial backgrounds, including the United States, Australia, China, England, Germany, India, Italy, the Netherlands, Pakistan and Turkey.^34^It was found that the primary source of test anxiety was exam grades.^35^ Severe anxiety symptoms were reported to occur in anticipation of and/or during professional licensing examinations, particularly those that contained test questions in a multiple-choice format.^34^ Therefore, the summative characteristics of the Saudi college assessment program may have led students to experience adverse cognitive and emotional effects, including impaired attention, problems with focusing and difficulties with the retrieval of stored knowledge.^36^ These problems, in turn, may have adversely affected their study habits,^37^ leading to a more superficial approach to learning.The SMS students were subjected to less frequent summative assessment but to more formative assessment compared to KSAU-HS, COM students. It appeared that the balance between summative and formative assessment in the SMS program reduced the negative anxiety effects of summative assessment. Moreover, the possible influence of cultural factors in enabling SMS students to cope with this anxiety and its subsequent effect on their perception of the assessment process cannot be ruled out. The assessment program in SMS may have created just the right degree of stress, leading to focused attention, improved memory^38^ and better overall results. Our data suggest that student perceive stress from summative exams as beneficial to a certain degree, beyond which it has negative effects. Therefore, negative stress resulting from an over focus on summative assessment should be balanced with adequate frequency of formative feedback.Both groups of students and supervisors recognized the importance and positive impact of formative assessment on students' learning. Despite this agreement, it appears that the presence of cultural differences between the two groups influenced students' responses to negative feedback. KSAU-HS, COM students' self-perception, emotions and professional culture have affected their ability to accept and accommodate supervisors’ negative feedback.^39^^, ^^40^Curriculum alignment of learning objectives, assessment and teaching and learning activities help students to achieve broad and direct exposure to core educational concepts.^41^ However, many students were actually selecting their own study objectives, thereby creating a hidden curriculum.^13^ The prevalence of this phenomenon among SMS students may have been a result of the less structured and formative nature of assessment compared with KSAU-HS, COM assessment program. KSAU-HS, COM assessment program, with its frequent summative assessments and highly structured format, was more successful in stimulating curriculum objective-based learning and in reducing the tendency to create a hidden curriculum. However, the emphasis on structured summative assessment has led to more anxiety and a tendency towards a superficial and achievement motivation study strategies.In fact, the health-education system in Saudi Arabia may have contributed to the development of such attitudes among KSAU-HS, COM students. A student’s prospects for residency training in Saudi Arabia are greatly dependent on their accumulative assessment grades. Consequently, students develop a very competitive attitude with the goal of achieving high scores, and this attitude makes them less likely to gamble in selecting their study objectives.There were similarities in students’ perceptions of clinical assessment as opposed to written assessment in between the two studied groups. Students particularly appreciated work based assessment that was conducive to learning and held significant value for them. Work-based assessment was perceived by the students as leading to more skilled doctors and was a stimulant for better approaches to learning. When students begin clinical training, they encounter different supervised learning environments and different assessment programs. In these learning environments, supervisors’ knowledge, skills, encouragement of a problem-solving approach, critical reflection on practice, supervision and assessment methods are perceived as important factors affecting students’ study strategies.^42^The results of this study regarding the importance of available time for teaching and clinical assessment are also confirmed by other studies.^43^^, ^^44^ Variations between institutions and supervisors in the acceptance of responsibility for clinical teaching and time allocated for supervision have been reported. In this study, limited time and resources for clinical teaching were regarded as barriers to high-quality teaching performance. This result was not affected by the various differences between the two groups.

### Study Limitations

One of the limitations of this study is related to the study population; at KSAU-HS, COM the student population is entirely male, whereas SMS has a mixed population of male and female students. A second limitation lies in the difficulty of comparing two very different groups. Additional cultural factors may have contributed to differences in study strategies, for example the differences between the multi-cultural society of Sydney and the ethnically homogenous Saudi student population at KSAU-HS, COM. Additional contextual factors could include the length of supervisors’ teaching experience. Furthermore, despite the fact that similar curricula were implemented in both schools, we cannot deny the possibility of curricular differences.

## Conclusions

Differences in assessment methods appear to lead to different perceptions and learning approaches; this might be mediated by some differences in cultural values. To maximize the educational impact of assessment programs and to avoid the possible negative effects of cultural barriers, a combination of formative and summative assessment is needed accompanied with precisely written curriculum objectives. There should be a balance between summative and formative assessment to stimulate stress, which helps students focus their attention, improve their performance and avoid the creation of a hidden curriculum. Such assessment programs should be tailored for each institution, taking into consideration not only assessment factors but also cultural values, preferences, health-education systems and job opportunities. Furthermore, students and supervisors should be prepared prior to the implementation of formative assessment through adequate orientation and faculty development programs. Such preparation will allow students to accommodate and benefit from negative feedback. Finally, there is a paucity of research in cross-cultural teaching and learning in a medical education context and we recommend further research that focuses on the cultural role on students’ perception of their assessment and the resulting study strategies.
